# Sex-Specific Association between Serum Uric Acid and Nonalcoholic Fatty Liver Disease in Type 2 Diabetic Patients

**DOI:** 10.1155/2016/3805372

**Published:** 2016-06-12

**Authors:** Nengguang Fan, Lijuan Zhang, Zhenhua Xia, Liang Peng, Yufan Wang, Yongde Peng

**Affiliations:** ^1^Department of Endocrinology and Metabolism, Shanghai First People's Hospital, Shanghai Jiao Tong University, Shanghai 200080, China; ^2^Department of Endocrinology, Shanghai Songjiang Center Hospital, Shanghai 201600, China; ^3^Department of Laboratory Medicine, Shanghai Songjiang Center Hospital, Shanghai 201600, China

## Abstract

Across-sectional study was performed in 541 type 2 diabetic patients to determine the relationship between serum uric acid (SUA) and NAFLD in type 2 diabetic patients. Clinical parameters including SUA were determined and NAFLD was diagnosed by ultrasonography. SUA was significantly higher in type 2 diabetic subjects with NAFLD than in those without NAFLD in men, but not in women. Furthermore, the prevalence rate of NAFLD increased progressively across the sex-specific SUA tertiles only in men (37.9%, 58.6%, and 72.6%, resp., *P* for trend < 0.001). After adjusting for confounding factors, the odd ratios (95% CI) for NAFLD were 1 (reference), 2.93 (95%CI 1.25–6.88), and 3.93 (95% CI 1.55–9.98), respectively, across the tertiles of SUA in men. Contrastingly, SUA levels in women were not independently associated with the risk of NAFLD. Our data suggests that SUA is specifically associated with NAFLD in male type 2 diabetic subjects, independent of insulin resistance and other metabolic factors.

## 1. Introduction

Nonalcoholic fatty liver disease (NAFLD), defined as the presence of hepatic steatosis in the absence of alcohol use and other causes of liver disease, has become one of the most prevalent liver diseases worldwide [1]. It represents a spectrum of conditions from simple steatosis to nonalcoholic steatohepatitis (NASH) and cirrhosis. A growing body of evidence has indicated a close relationship between NAFLD and obesity, dyslipidemia, diabetes, and insulin resistance [1, 2]. For this reason, NAFLD is considered to be a hepatic manifestation of metabolic syndrome [2].

Serum uric acid (SUA) is the end-product of purine nucleotide catabolism and maintained by the balance between uric acid production and excretion. In the past decade, SUA has shown to be closely related to components of metabolic syndrome, including obesity, dyslipidemia, and diabetes [3–6]. Individuals with increased SUA are at higher future risk of metabolic syndrome, type 2 diabetes, and cardiovascular diseases independent of other known risk factors, suggesting a potential role of SUA in the pathogenesis of these diseases [7–10].

Recently, the relationship between SUA and NAFLD has also been clarified. SUA levels were significantly higher in individuals with NAFLD than in those without NAFLD, and the prevalence rate of NAFLD was progressively increased in parallel to the increment of SUA [11–13]. In prospective studies, elevation of SUA was shown to be independently associated with higher incidence of NAFLD after adjusting for potential confounders [14, 15]. Previously, the association between SUA and NAFLD has primarily been investigated in general population [11, 12, 14], also in pre- and postmenopausal women, and in nondiabetic subjects [16, 17]. However, whether the relationship between SUA and NAFLD also exists in type 2 diabetic subjects, who have a high incidence rate of NAFLD, has not been investigated yet.

In the present study, we performed a cross-sectional investigation to determine whether SUA is associated with NAFLD in type 2 diabetic subjects.

## 2. Subjects and Methods

### 2.1. Subjects

All type 2 diabetic patients were recruited from the Department of Endocrinology and Metabolism in Shanghai First People's Hospital between May 2013 and June 2014. The diagnosis of type 2 diabetes was defined according to the 1999 World Health Organization criteria. All participants were requested to complete a standardized questionnaire that included questions on the history of present and past illnesses and medical therapies. Subjects with an alcohol intake > 140 g/week for men and 70 g/week for women, a history of viral hepatitis, autoimmune hepatitis, or other forms of chronic liver disease, and a history of heart failure or renal diseases and those taking medications affecting SUA were excluded from the study. Finally, a total of 541 type 2 diabetic patients were included in analysis. This study was approved by the Institutional Review Board of Shanghai First People's Hospital affiliated to Shanghai Jiao Tong University School of Medicine and performed in accordance with the principle of Helsinki Declaration II. Written informed consent was obtained from all subjects.

### 2.2. Anthropometric and Biochemical Measurements

All subjects were assessed after overnight fasting for at least 10 h. Body weight, height, and systolic and diastolic blood pressure (SBP, DBP) were measured by an experienced physician. BMI was calculated as body weight in kilograms divided by body height squared in meters.

Blood samples were collected by one experienced nurse. Fasting serum triglycerides (TG), total cholesterol (TC), low-density lipoprotein cholesterol (LDL-C), high-density lipoprotein cholesterol (HDL-C), alanine aminotransferase (ALT), aspartate aminotransferase (AST), serum creatinine (Scr), and insulin were measured using an autoanalyzer (Beckman, Palo Alto, CA). Blood glucose was measured with glucose oxidase method. HbA1c was determined by high-performance liquid chromatography. Homeostasis model assessment of insulin resistance (HOMA-IR) was calculated as fasting insulin (mIU/L) × fasting glucose (mmol/L)/22.5. The abbreviated Modification of Diet in Renal Disease (MDRD) formula recalibrated for Chinese was used to estimate glomerular filtration rate (eGFR): 186 × [Scr × 0.011]^−1.154^ × [age]^−0.203^ × [0.742  if  female] × 1.233, where Scr is serum creatinine expressed as *μ*mol/L and 1.233 is the adjusting coefficient for Chinese.

### 2.3. Diagnosis of NAFLD

The diagnosis of NAFLD was based on the results of abdominal ultrasonography by a trained ultrasonographer using a high-resolution B-mode tomographic ultrasound system with a 3.5-MHz probe (Toshiba, Tokyo, Japan). According to Diagnostic Criteria of Nonalcoholic Fatty Liver Disease by the Chinese Society of Hepatology in 2010, hepatic steatosis was defined by the presence of at least 2 of 3 of the following abnormal findings: diffuse hyperechogenicity of the liver relative to the kidneys; attenuation of the ultrasound beam; and poor visualization of intrahepatic architectural details [18]. Alcohol consumption or viral or autoimmune liver disease was excluded before NAFLD diagnosis.

### 2.4. Statistical Analysis

All statistical analyses were performed using SPSS 13.0 (Chicago, IL). Continuous variables were presented as means ± SD or median (interquartile range), and categorical variables were displayed as percentages (%). Non-normally distributed data were logarithmically transformed before analysis. Differences between two groups were tested by Student's *t*-test for continuous variables and *χ* ^2^ test for categorical variables. Pearson's correlations were performed to evaluate the associations between SUA and other metabolic risk factors, as well as multivariate stepwise linear regression model to identify the independent factors related to SUA. Logistic regression was also used to evaluate the association between SUA and NAFLD. The sex-specific cutoff points of SUA tertiles were as follows: tertile 1: ≤265.6 (men) and ≤232.0 (women) *μ*mol/L; tertile 2: 265.6–338.4 (men) and 232.0–299.4 (women) *μ*mol/L; and tertile 3: ≥338.4 (men) and ≥299.4 (women) *μ*mol/L. *P* < 0.05 was considered statistically significant.

## 3. Result

### 3.1. Clinical Characteristics of the Study Population

Among the 541 type 2 diabetic patients, 270 were women and 271 were men. The overall prevalence rate of NAFLD was 56.6%, and no difference was observed between women and men (56.7% versus 56.5%, *P* > 0.05). SUA was significantly higher in men than in women (311.6 ± 90.4 versus 270.1 ± 84.3 *μ*mol/L, *P* < 0.001); we therefore performed the following analyses separately in men and women.

Clinical and biochemical characteristics of the study population according to the presence of NAFLD were summarized in Table 1. Subjects with NAFLD had a shorter duration of diabetes, higher levels of BMI, FPG, TG, ALT, AST, and HOMA-IR, and lower levels of HDL-C as compared with those without NAFLD in both genders (all *P* values < 0.05). In contrast, significantly higher levels of SUA were only observed in men, but not in women, upon comparing the subjects with and without NAFLD (334.1 ± 91.3 versus 281.1 ± 80.3 *μ*mol/L, *P* < 0.001, in men; 275.7 ± 80.1 versus 267.0 ± 88.2 *μ*mol/L, *P* = 0.403, in women). No significant difference was found in SBP, HbA1C, TC, LDL-C, eGFR, and insulin use between the two groups.

### 3.2. Associations between SUA and Metabolic Risk Factors

Next, the associations between SUA and other metabolic risk factors were further investigated in both genders. In women, analysis of Pearson's correlation showed that SUA was positively associated with BMI, SBP, and TG, while it was negatively associated with HDL-C, FPG, HbA1C, and eGFR (Table 2). Multiple stepwise linear regression analysis further revealed that BMI and eGFR were two independent determiners of SUA in female type 2 diabetic subjects (Table 2). In men, SUA was also positively associated with BMI, while it was inversely correlated with HDL-C, HbA1C, and eGFR (Table 2). Similar to women, BMI and eGFR were independently associated with SUA in men.

### 3.3. Associations of SUA with NAFLD

**Figure 1:**
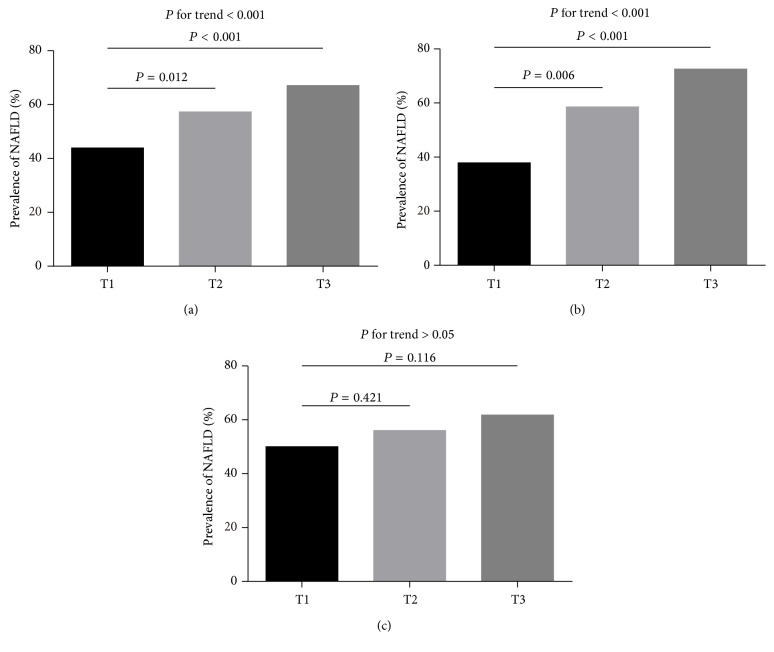
Prevalence of NAFLD according to tertiles of SUA. T1, T2, and T3 represent tertile 1, tertile 2, and tertile 3 of SUA in total subjects (a), in men (b), and in women (c), respectively.

All of the study subjects were divided into 3 groups according to the sex-specific tertiles of SUA and the prevalence of NAFLD was investigated. As shown in Figure 1(a), the prevalence of NAFLD was progressively increased from the lowest tertile across to the highest one of SUA tertiles (43.9%, 57.3%, and 67.1%, resp., *P* for trend < 0.001). When stratified by gender, the prevalence of NAFLD in men was also significantly increased across the SUA tertiles (37.9%, 58.6%, and 72.6%, resp., *P* for trend < 0.001). Strikingly, a significant increment in NAFLD prevalence was observed in the second and third tertiles as compared with the lowest one (Figure 1(b)). In contrast, the prevalence rate of NAFLD was not significantly different across the tertiles of SUA in women (50.0%, 56.0%, and 61.8%, resp., *P* for trend > 0.05, Figure 1(c)).

Logistic regression analysis was further performed to determine the association between SUA tertiles and risk of NAFLD. As shown in Table 3, SUA tertiles in women were not associated with the risk of NAFLD in both univariate and multivariate analyses. In men, univariate analysis revealed increased odd ratios (ORs) for NAFLD across SUA tertiles (model 1). After adjustment for duration of diabetes, age, BMI, SBP, and DBP (model 2), the ORs of NAFLD for increasing serum UA tertiles were 1 (reference), 1.69, and 2.12 (*P* for trend = 0.016). With further adjustment for insulin and OADs use, FPG, HbA1c, TG, TC, HDL-C, LDL-C, eGFR, and HOMA-IR (model 3), the ORs for NAFLD in the 2nd and 3rd tertiles were 2.93 and 3.93, respectively (*P* for trend = 0.003).

## 4. Discussion

NAFLD has been the most common liver disease worldwide, especially in obesity and type 2 diabetic patients. Recent evidence showed a close relationship between SUA and NAFLD in several populations. However, the association of them has not been investigated yet in type 2 diabetic patients. In the present study, we found that SUA was specifically associated with NAFLD in male type 2 diabetic patients, not in female participants. To our current knowledge, this is the first report to explore the association between SUA and NAFLD in type 2 diabetic subjects.

Both SUA and NAFLD are closely associated with obesity and related metabolic abnormalities. Recently, the relationship between them has also been revealed. SUA was increased in NAFLD subjects and independently correlated with the risk of NAFLD in both cross-sectional and prospective studies in several populations [11, 14, 15]. More recently, a large population-based study presented a novel finding that the association between SUA and NAFLD was significantly greater in females than in males [19]. On the contrary, our present study in type 2 diabetic subjects showed significantly higher SUA levels in NAFLD group than in non-NAFLD group in males, not in females. Moreover, elevation of SUA was independently associated with higher risk of NAFLD only in male type 2 diabetic patients. Although the difference of the study populations may be responsible for the above inconsistency, the reasons for sex-specific association between SUA and NAFLD remain to be determined. One previous study showed that SUA was related to NAFLD in postmenopausal but not premenopausal women [17], suggesting a potential role of female hormones in attenuating the association of SUA with NAFLD. Due to the lack of data on menstruation, we could not determine whether the association between SUA and NAFLD in females is also modified by menstruation in the present study. Future studies are needed to confirm sex-specific relationship between SUA and NAFLD. Moreover, in addition to type 2 diabetic patients, the association between SUA and NAFLD merits investigation in other populations with metabolic disorders such as metabolic syndrome.

The potential mechanism linking SUA to NAFLD is not fully established. In fact, SUA is related to obesity and hypertriglyceridemia, which are risk factors for NAFLD. Our study also documented that SUA was positively associated with BMI and triglycerides. However, the association between SUA and NAFLD remained significant after controlling for BMI and triglycerides, excluding the possibility that they mediate the association between SUA and NAFLD. Another potential mechanism that explains the relationship between them is insulin resistance. Uric acid was found to directly inhibit insulin signaling and induce insulin resistance [20], which is considered to be an essential mechanism of NAFLD [21]. Interventions that ameliorate insulin resistance could lead to improvement of fatty liver [21–23]. Nevertheless, after adjustment for insulin resistance in the present study, the association of SUA with NAFLD was still significant. Thus, we could assume that there may be other mechanisms involved in such association.

Recently, evidence from basic researches indicated a direct role of uric acid in the pathogenesis of NAFLD. Lanaspa et al. showed that uric acid induced hepatic steatosis in vitro by generating mitochondrial oxidative stress [24], while, in another study, uric acid was found to stimulate fat accumulation via generation of endoplasmic reticulum stress and SREBP-1c activation in hepatocytes [25]. More recently, it was revealed that uric acid regulated hepatic steatosis through the NLRP3 inflammasome-dependent mechanism [26]. However, the above in vitro evidence remains to be confirmed in vivo including animal and clinical studies.

Our study adds evidence to the association between SUA and NAFLD. Together with a potential role of SUA in the pathogenesis of NAFLD, Sun et al. have put forward that reduction of SUA may be a promising potential treatment for patients with NAFLD [27]. In fact, hypouricemic therapies have been shown to significantly ameliorate hepatic steatosis in obese mice [28]. However, the effect of attenuation of SUA on NAFLD remains to be investigated in human NAFLD.

There are several limitations that require consideration. First, population of the present study was relatively small. Hence, the association between SUA and NAFLD should be confirmed in studies with a larger sample. Second, our study was cross-sectional, which did not allow making a cause-effect inference. Third, the best method for an accurate diagnosis of NAFLD is liver biopsies. Ultrasonic examination, which was applied in the present study for diagnosis of NAFLD, is not sensitive enough to detect mild liver steatosis. Moreover, due to lack of the quantification of the severity of NAFLD based on hepatic ultrasound, relationship between SUA and hepatic steatosis severity could not be evaluated. However, this noninvasive method is still widely used in clinical practice and epidemiological studies and is accepted for its sensitivity and specificity in detecting hepatic steatosis.

In conclusion, the present study showed a sex-specific association between SUA and NAFLD in type 2 diabetic patients. The study added more evidence to the hypothesis that elevation of SUA increases the risk of NAFLD and may be helpful for early treatment of NAFLD in type 2 diabetic patients.

## Figures and Tables

**Table 1:** Clinical and biochemical characteristics of the study subjects.

Characteristics	Women			Men		
	Non-NAFLD	NAFLD	*P* value	Non-NAFLD	NAFLD	*P* value
*n*	117	153		118	153	
Age (years)	61.7 ± 13.0	59.6 ± 10.2	0.163	57.7 ± 13.7	53.9 ± 15.1	0.032
Duration (years)	7 (3–11)	3 (0–9)	<0.001	5 (0–10)	2 (0–6)	0.041
BMI (kg/m^2^)	22.8 ± 3.4	26.1 ± 3.6	<0.001	23.0 ± 2.9	26.1 ± 3.5	<0.001
SBP (mmHg)	134.4 ± 18.5	136.6 ± 18.3	0.335	133.1 ± 18.8	134.1 ± 17.3	0.634
DBP (mmHg)	79.4 ± 10.0	82.8 ± 10.8	0.009	81.6 ± 9.9	83.1 ± 11.4	0.242
FPG (mM)	9.2 ± 4.2	11.0 ± 3.5	<0.001	9.8 ± 3.5	11.1 ± 3.0	0.001
HbA1C (%)	10.4 ± 7.2	9.9 ± 2.3	0.49	10.3 ± 2.5	10.3 ± 2.1	0.99
TG (mM)	1.2 (0.9–1.6)	1.7 (1.3–2.5)	<0.001	1.2 (0.9–1.6)	1.8 (1.2–3.0)	<0.001
TC (mM)	4.8 ± 1.3	5.0 ± 1.2	0.204	4.6 ± 1.2	4.7 ± 1.4	0.4
LDL-C (mM)	3.1 ± 1.0	3.2 ± 1.1	0.171	3.0 ± 1.0	2.9 ± 1.0	0.652
HDL-C (mM)	1.5 ± 0.5	1.2 ± 0.3	<0.001	1.3 ± 0.4	1.1 ± 0.4	<0.001
ALT (IU/L)	9 (7–11)	13 (9–23)	<0.001	10 (8–14)	14 (10–23)	<0.001
AST (IU/L)	17 (15–20)	21 (16–29)	<0.001	17 (14–21)	20 (16–28)	<0.001
eGFR (mL/min/1.73 m^2^)	132 (112–160)	142 (116–170)	0.11	140 (112–161)	137 (115–167)	0.661
HOMA-IR	2.4 (1.5–4.1)	4.5 (3.0–7.2)	<0.001	2.4 (1.6–3.8)	3.9 (2.6–6.0)	<0.001
SUA (*μ*M)	267.0 ± 88.2	275.7 ± 80.1	0.403	281.1 ± 80.3	334.1 ± 91.3	<0.001
Insulin therapy (%)	24.1	15.6	0.238	9.8	12.9	0.604
OADs therapy (%)	48.3	25	0.008	33.3	34.3	0.913

Continuous variables were presented as means ± SD or median (interquartile range), and categorical variables were displayed as percentages (%). OADs, oral antidiabetic drugs.

**Table 2:** Pearson's correlation and stepwise regression analysis of determinants of SUA.

	Women				Men			
	*r*	*P*	Standardized *β*	*P*	*r*	*P*	Standardized *β*	*P*
Age (years)	0.083	0.159	—	—	0.013	0.826	—	—
Duration	0.035	0.55	—	—	−0.017	0.779	—	—
BMI (kg/m^2^)	0.228	<0.001	0.195	0.001	0.399	<0.001	0.361	<0.001
SBP (mmHg)	0.165	0.005	—	—	0.055	0.356	—	—
DBP (mmHg)	0.085	0.148	—	—	0.041	0.496	—	—
TG (mM)	0.117	0.049	—	—	0.115	0.056	—	—
TC (mM)	0.026	0.657	—	—	0.022	0.722	—	—
LDL-C (mM)	−0.011	0.859	—	—	−0.026	0.67	—	—
HDL-C (mM)	−0.143	0.016	—	—	−0.165	0.006	—	—
FPG (mM)	−0.141	0.017	—	—	−0.047	0.434	—	—
HbA1C (%)	−0.117	<0.054	—	—	−0.185	0.003	—	—
eGFR (mL/min/1.73 m^2^)	−0.422	<0.001	−0.221	0.035	−0.285	<0.001	−0.348	<0.001
HOMA-IR	0.008	0.89	—	—	−0.013	0.824	—	—

**Table 3:** The risk of prevalent NAFLD according to tertiles of SUA.

	Women				Men			
	Tertile 1	Tertile 2	Tertile 3	*P* for trend	Tertile 1	Tertile 2	Tertile 3	*P* for trend
Model 1	1	1.28 (0.71–2.30)	1.62 (0.89–2.95)		1	2.32 (1.26–4.26)	4.34 (2.27–8.28)	
*P* value		0.421	0.117	0.117		0.007	<0.001	<0.001

Model 2	1	0.95 (0.49–1.85)	0.94 (0.47–1.88)		1	1.69 (0.90–3.57)	2.12 (1.18–5.24)	
*P* value		0.89	0.851	0.849		0.099	0.017	0.016

Model 3	1	0.99 (0.46–2.12)	1.08 (0.45–2.62)		1	2.93 (1.25–6.88)	3.93 (1.55–9.98)	
*P* value		0.975	0.864	0.871		0.014	0.004	0.003

Data are odds ratios (95% confidence interval) compared with tertile 1 group. Participants without NAFLD are defined as 0 and those with NAFLD are defined as 1.

Model 1 is unadjusted.

Model 2 is adjusted for age, duration of diabetes, SBP, DBP, and BMI.

Model 3 is further adjusted for insulin and OADs therapy, FPG, HbA1C, TG, TC, LDL-C, HDL-C, eGFR, and HOMA-IR.
